# Developing virtual and augmented reality applications for science, technology, engineering and math education

**DOI:** 10.2144/btn-2023-0029

**Published:** 2023-06-09

**Authors:** Christopher L Hemme, Rachel Carley, Arielle Norton, Moez Ghumman, Hannah Nguyen, Ryan Ivone, Jyothi U Menon, Jie Shen, Matthew Bertin, Roberta King, Elizabeth Leibovitz, Roy Bergstrom, Bongsup Cho

**Affiliations:** 1 Rhode Island IDeA Network of Biomedical Research Excellence (RI-INBRE); 2 College of Pharmacy, University of Rhode Island, Kingston, RI 02881, USA; 3 College of Engineering, University of Rhode Island, Kingston, RI 02881, USA; 4 Information Technology Services, Innovative Learning Technologies Program, University of Rhode Island, Kingston, RI 02881, USA

**Keywords:** augmented reality, biomedical sciences, pharmaceutical sciences, STEM education, virtual reality

## Abstract

The Rhode Island IDeA Network of Biomedical Research Excellence Molecular Informatics Core at the University of Rhode Island Information Technology Services Innovative Learning Technologies developed virtual and augmented reality applications to teach concepts in biomedical science, including pharmacology, medicinal chemistry, cell culture and nanotechnology. The apps were developed as full virtual reality/augmented reality and 3D gaming versions, which do not require virtual reality headsets. Development challenges included creating intuitive user interfaces, text-to-voice functionality, visualization of molecules and implementing complex science concepts. In-app quizzes are used to assess the user's understanding of topics, and user feedback was collected for several apps to improve the experience. The apps were positively reviewed by users and are being implemented into the curriculum at the University of Rhode Island.

## Method summary

Virtual/augmented reality apps were developed in Unity to teach topics in biomedical science. In-app 3D models were created to provide a scientifically accurate user experience. Apps were tested on High Tech Computer Vive-style virtual reality headsets.

## Graphical abstract

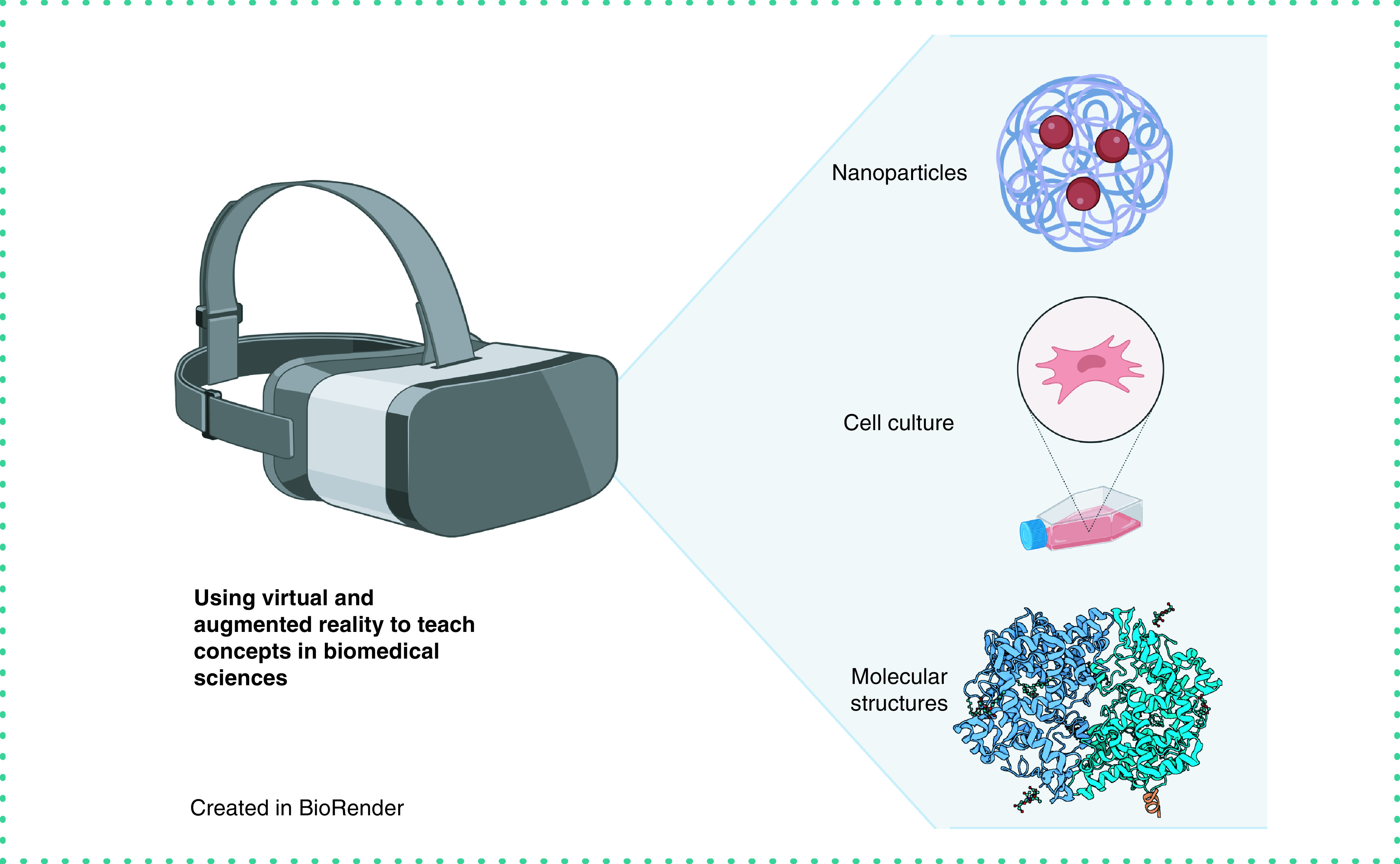

Virtual reality (VR) technology has existed since the 1970s but has only recently advanced to the point of being commercially viable for everyday applications [1–3]. Physical reality and VR represent end points on the reality–virtuality continuum (Figure 1), with mixed reality (MR) representing the merging of different degrees of virtual and real content [4]. MR includes augmented reality (AR; virtual content overlaid in the real world) and augmented virtuality (AV; real content integrated into a virtual world). Developers create applications that lie on this spectrum. Developers must consider cost, device computing power, ease of use and desired immersiveness when designing applications. Each application has unique requirements that developers must consider [5,6].

**Figure 1.**
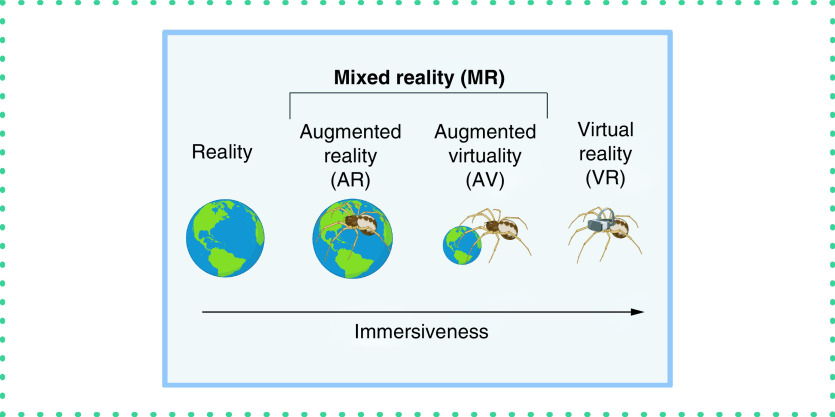
Reality–virtuality spectrum. The reality–virtuality spectrum is a simplified diagram of virtuality spanning from true reality (left) to fully immersive virtual reality (right). Between the two lies mixed reality, which ranges from overlaying virtual content onto the real world (augmented reality) to inserting real-world content into a virtual world (augmented virtuality). Figure created in BioRender.

While public experience with MR applications is primarily through gaming, the technology is increasingly being used by academics and researchers for science, technology, engineering and mathematics (STEM) purposes [1–3,7–9]. Virtual content allows interactivity that is not possible in physical reality or on a computer screen. For example, virtual rendering of protein structures provide a stereoscopic model of the molecule that can be intuitively manipulated. App-based training can be offered to large numbers of students simultaneously, which may not be possible in the real world due to insufficient workspaces and resources. Numerous VR/AR/MR applications have been developed for the biomedical sciences, including apps for molecular visualization [9–15], rehabilitation [15–18], data visualization [19–23] and general STEM education [8,17,24,25].

The Rhode Island IDeA Network of Biomedical Research Excellence (RI-INBRE) [26] Molecular Informatics Core (MIC) provides data science services to the RI-INBRE network. In cooperation with the University of Rhode Island College of Pharmacy (URI COP), the MIC maintains VR equipment for use by instructors, researchers and students. Initially, the use of this equipment focused on commercial or public apps used to teach concepts in biomedical science, including Nanome [10] and ChimeraX (molecule visualization) [13] and ConfocalVR (confocal microscopy imaging) [19]. However, it soon became apparent that to fully utilize the potential of the VR hardware, custom applications needed to be developed and tailored to specific scientific topics.

The MIC collaborates with the URI Information Technology Services Innovative Learning Technologies (ILT) program to develop such apps. Since 2007, the ILT program has worked with the COP 3D Science Visualization Teaching Program (https://web.uri.edu/pharmacy/3d/) directed by Bongsup Cho. The projects began with the 3D Animation Program and 3D Projection and were expanded in 2011 with 3D animation, projection and printing with support from the Champlin Foundation, a RI-based philanthropic organization. The earlier COP-ILT animation project involved COP faculty members, pharmacy students and computer science undergraduates in creating short video clips illustrating how drugs work at the molecular and cellular levels. These efforts have been transformative, and the 3D animation program has drawn nearly 18 million views on the *URIanimation* YouTube channel.

Here, VR/AR and 3D gaming apps developed by the RI-INBRE/ILT/COP collaboration to teach concepts in pharmacy, medicinal chemistry and structural biology are described. Completed versions of several of these apps are available.

## Materials & methods

The apps are designed in Unity, which is widely used in the gaming industry because of its ease of use and customizability. The apps were developed for High Tech Computer (HTC, Taiwan) -style headsets (Vive, Cosmos, etc.) or Android devices. Blender was primarily used for generating 3D models. UnityMol [27] was used for processing and visualizing molecular structures, with structures obtained from PubChem and/or the Research Collaboratory for Structural Bioinformatics (RCSB) Protein Data Bank (PDB). Cosmos headsets use Alienware M15 R2 laptops, which simplifies VR demo setup. The ILT program has a dedicated VR space in Tyler Hall. Each semester, the ILT receives applications for projects and time is budgeted (usually a year) for the development of the app.

## Results & discussion

### Year 1 apps

The RI-INBRE-COP-ILT collaboration began in 2018–2019. It stemmed from a desire by RI-INBRE to expand the scope of data science services offered, and by ILT to expand their existing data visualization skill set into VR. These apps were primarily proof-of-concept experiments to gain skills in VR app development and to assess the utility of VR in advancing the educational mission of COP.

#### Aspirin VR

**Figure 2.**
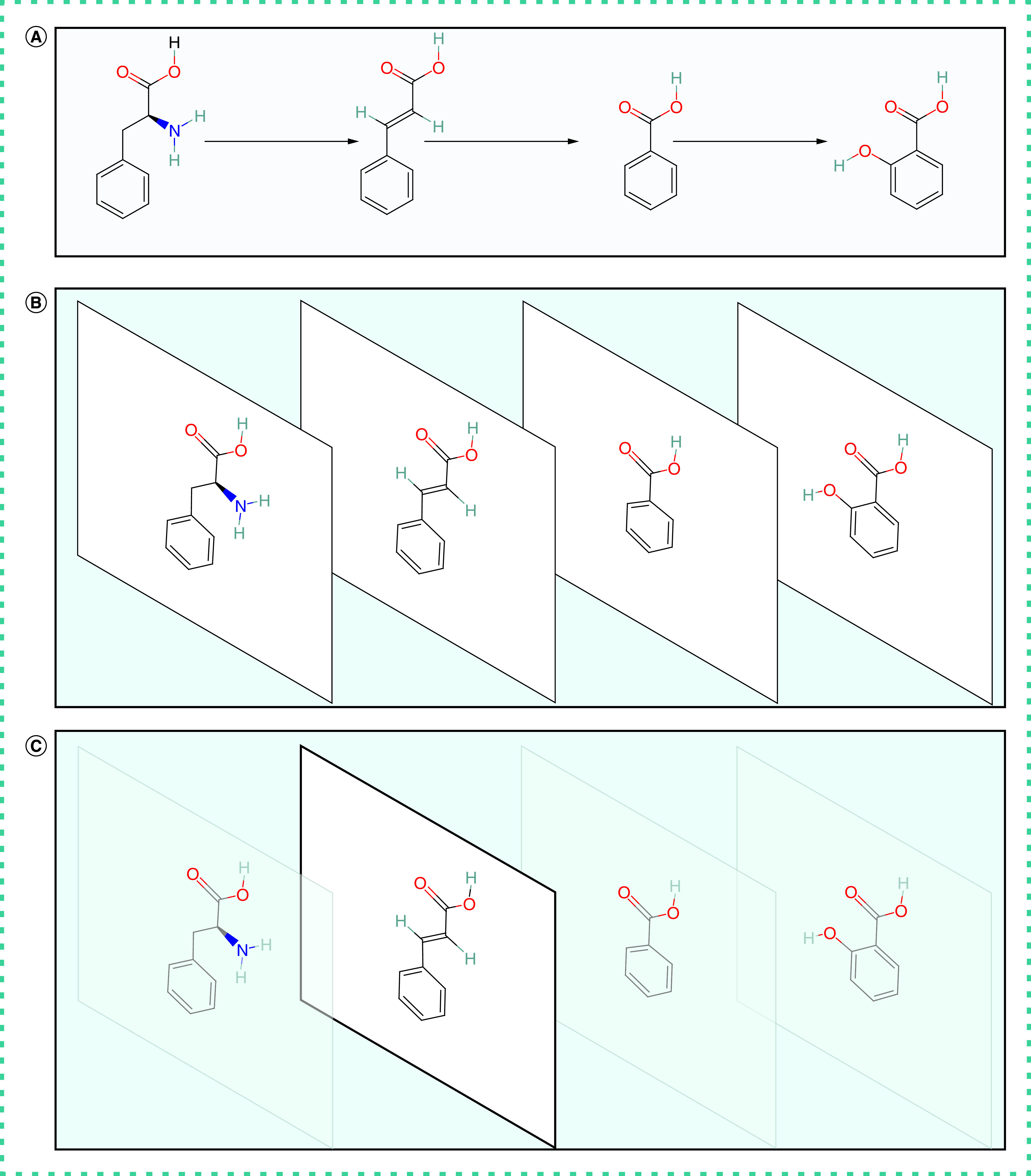
Molecular slideshow effect for visualizing biochemical pathways in virtual reality. **(A)** Biochemical pathways (e.g., salicylic acid biosynthesis) are traditionally visualized in two dimensions as a linear progression from initial substrates to final product. **(B)** To better visualize this process in virtual reality, the biochemical pathway is rotated into the third dimension, creating an image stack with a single molecule occupying each layer. The molecules are then resized, oriented and aligned by common atoms to produce a seamless transition between each layer. **(C)** A molecular slide show effect can be created by changing the visibility of each layer in succession creating the effect of a single molecule changing as it progresses along the pathway.

The first app to be developed focused on aspirin and its precursor, salicylic acid. The app was envisioned as a virtual museum consisting of five rooms: introduction (credits, tutorial for interacting with the environment); salicylic acid as a natural product; history and development of aspirin; clinical applications of aspirin and its alternatives; and pharmacology and biochemistry of aspirin and its COX-2 target. As this app was the first attempt at developing an app, challenges involving the user interface and how best to present the scientific content were encountered. For example, the usual left-to-right rendering of biochemical pathways proved to be unwieldy in VR, prompting alternative visualization strategies, for example, rotating the left-to-right pathway into the third dimension, then aligning and resizing the molecules so that common atoms overlap (Figure 2). Visualizing individual molecules creates the impression of a single molecule changing as it progresses through the pathway. An interactive quiz where molecular components of the pathway can be dragged and dropped in the appropriate location to advance the pathway were also used.

#### Methotrexate VR

**Figure 3.**
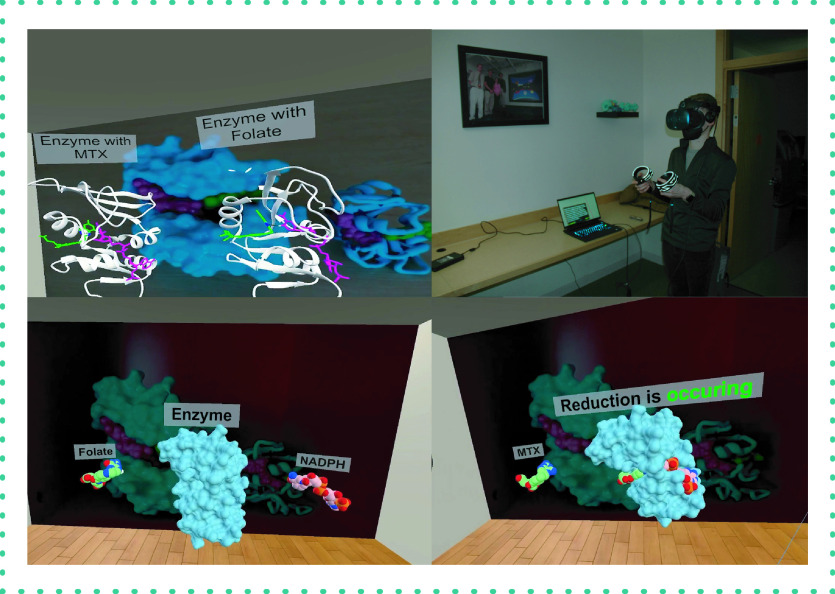
Methotrexate app. Virtual reality app demonstrating mechanism of action of anticancer drug, methotrexate. This app compares DHFR binding interactions for the natural substrate FA and the cancer drug MTX and its cofactor NADPH. The app shows the importance of the molecular spatial arrangement of MTX and FA that dictates the outcome of the DFHR's enzymatic reaction. For example, users see the proximity between the nicotine ring of NADPS (e.g., source of hydrogen) and the pteridine ring of FA, facilitating the reduction process. That's not the case with MTX. Students are instructed to identify the structural reasons why MTX blocks the DFHR enzymatic action, which explains its anticancer activity. With the app, users learn the importance of understanding small molecule-protein interactions and structural biology. FA: Folic acid; MTX: Methotrexate.

Anticancer drug methotrexate (MTX) is a folate inhibitor targeting the DHFR enzyme, resulting in interference with DNA synthesis and cellular repair. This app demonstrates the activity of MTX when interacting with the DHFR enzyme and its cofactor NADPH, and requires the user to interact with the molecules (Figure 3). This app was designed as a single virtual room focused on the activity of a single enzyme (DHFR), allowing experimentation with molecular animations and interactive quizzes that provided the user with a more detailed look at the mechanisms of action. Users saw how the spatial arrangement of the MTX molecule differs from the natural substrate folate and the activity of the enzyme with and without MTX inhibition and demonstrated how the NADPH cofactor reduces the folic acid substrate. This was the first app utilizing text-to-voice functionality to guide the user through the app.

#### Natural products VR

Students in Biomedical and Pharmaceutical Sciences (BPS) class BPS451, Techniques in Medicinal Chemistry and Molecular Biology, complete laboratory exercises and procedures related to drug discovery and analytical chemistry. Students harvest medicinal plants, test extracts in bioassays, fractionate bioactivity extracts using chromatographic procedures and identify bioactive components using HPLC and mass spectrometry (MS). Structure-activity relationships (SARs) are key in drug discovery and medicinal chemistry to make structural modifications to small-molecule drugs. Following a lecture on carmaphycin B (a natural product isolated from a marine cyanobacterium), students enter the “virtual laboratory” containing refrigerators, HPLC systems and pipettes. Students are given in-app quizzes and directed to the answers on the laboratory bench, consisting of chemical models. To answer each question, students place their selected molecule on a laboratory table, which turns green for a correct answer and red for an incorrect answer. The last question consists of the carmaphycin lead molecule posed in its receptor and students determine which drug–target interactions are responsible for binding. The integration of the journal article, the pre-VR lecture and the VR module are an innovative way to teach students drug development and lead optimization.

### Year 2 apps

In 2020, the current authors began creating more sophisticated apps and began experimenting with AR and 3D gaming versions of the apps accessible on desktops and laptops without the need for headsets. This was vital during the coronavirus 2019 (COVID-19) pandemic, when classes switched from in-person to virtual formats. In response to the pandemic, RI-INBRE initiated a new funding mechanism, the Enhanced Virtual Education, Research and Training (EVEREST) Pilot Project. Two RI-INBRE researchers, Jyothi Menon and Jie Shen from URI COP, initiated VR-based EVEREST projects, and these were the first apps to be developed as both VR and 3D gaming versions.

#### Medicinal garden AR app

**Figure 4.**
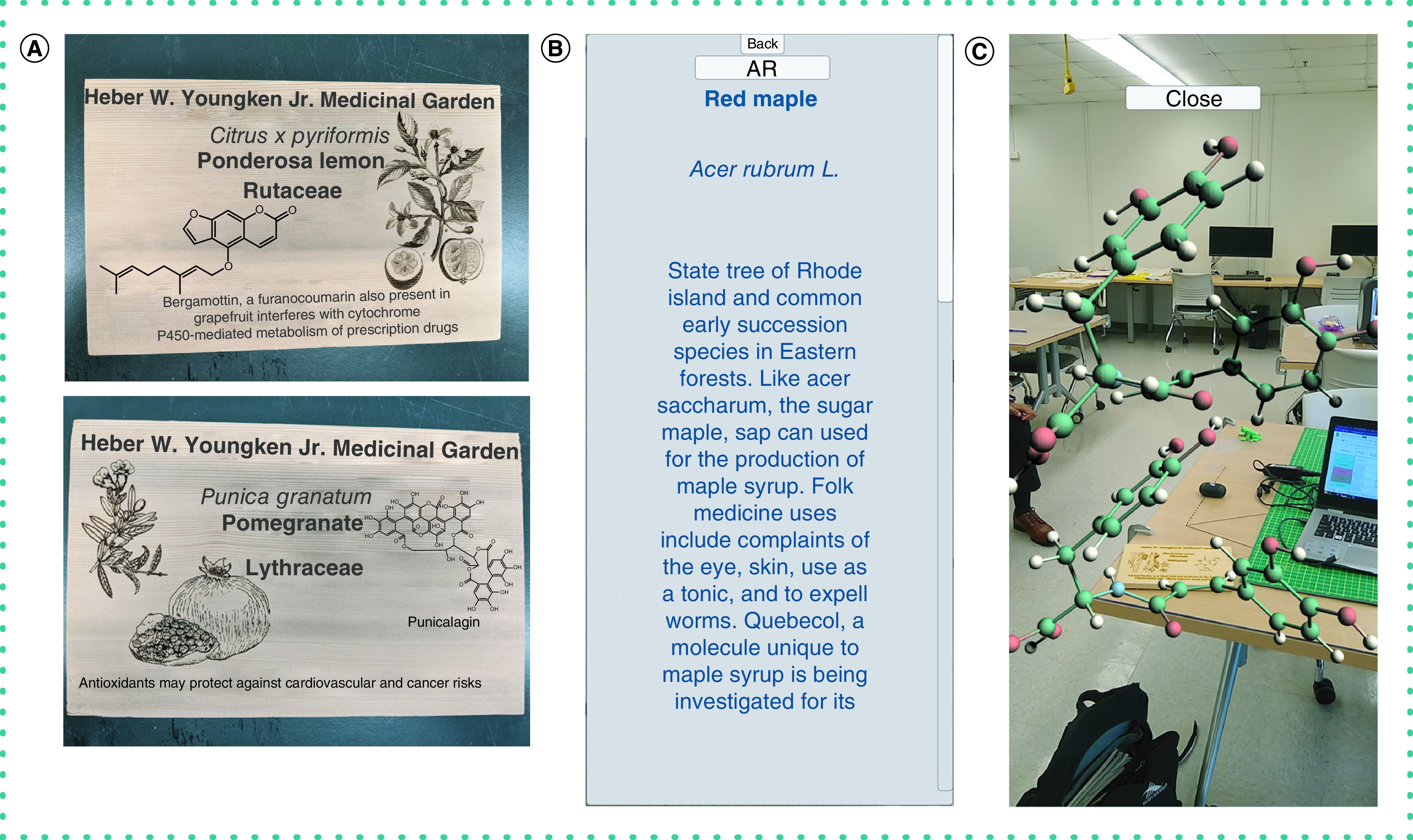
Augmented reality Medicinal Garden App. Augmented reality app usable in the Heber W. Youngkin Medicinal Garden at the University of Rhode Island. (A) An example of scannable wooden signs assigned to plants in the garden. Signs are scannable by mobile devices running the augmented reality app, which links to a local database containing information about the plants. **(B)** An example of the type of information assigned to each plant. Upon scanning the sign, the app displays information about the taxonomy, geographical range, toxicity and medicinal importance of each plant. **(C)** An example of a natural product associated with the plant in the database. Three relevant natural products/toxins are assigned to each plant and the molecule is displayed in augmented reality using UnityMol.

The COP maintains the Heber W. Youngkin Jr. Medicinal Garden containing >200 medicinal and >500 ornamental plants and is a key component of the medicinal chemistry curriculum. To enhance the Garden experience, an Android device-based AR app was developed to provide information about individual plants. AR allowed the overlay of virtual content on the garden with minimal need for physical alterations (e.g., scannable plant signs; Figure 4A). A total of 20 plants were initially chosen, and information was gathered including toxicity, geographical range and medicinal uses of the plants (Figure 4B). Structures of natural products were downloaded from PubChem and displayed in-app using UnityMol (Figure 4C).

#### Enhanced virtual education, research & training VR training apps

Two VR apps were developed through the EVEREST program. Both apps are designed as virtual laboratories where students can safely conduct complex experimental procedures. These were the first apps using custom 3D objects, allowing for the creation of visually accurate biomedical laboratory environments.

#### Cell culture training VR

**Figure 5.**
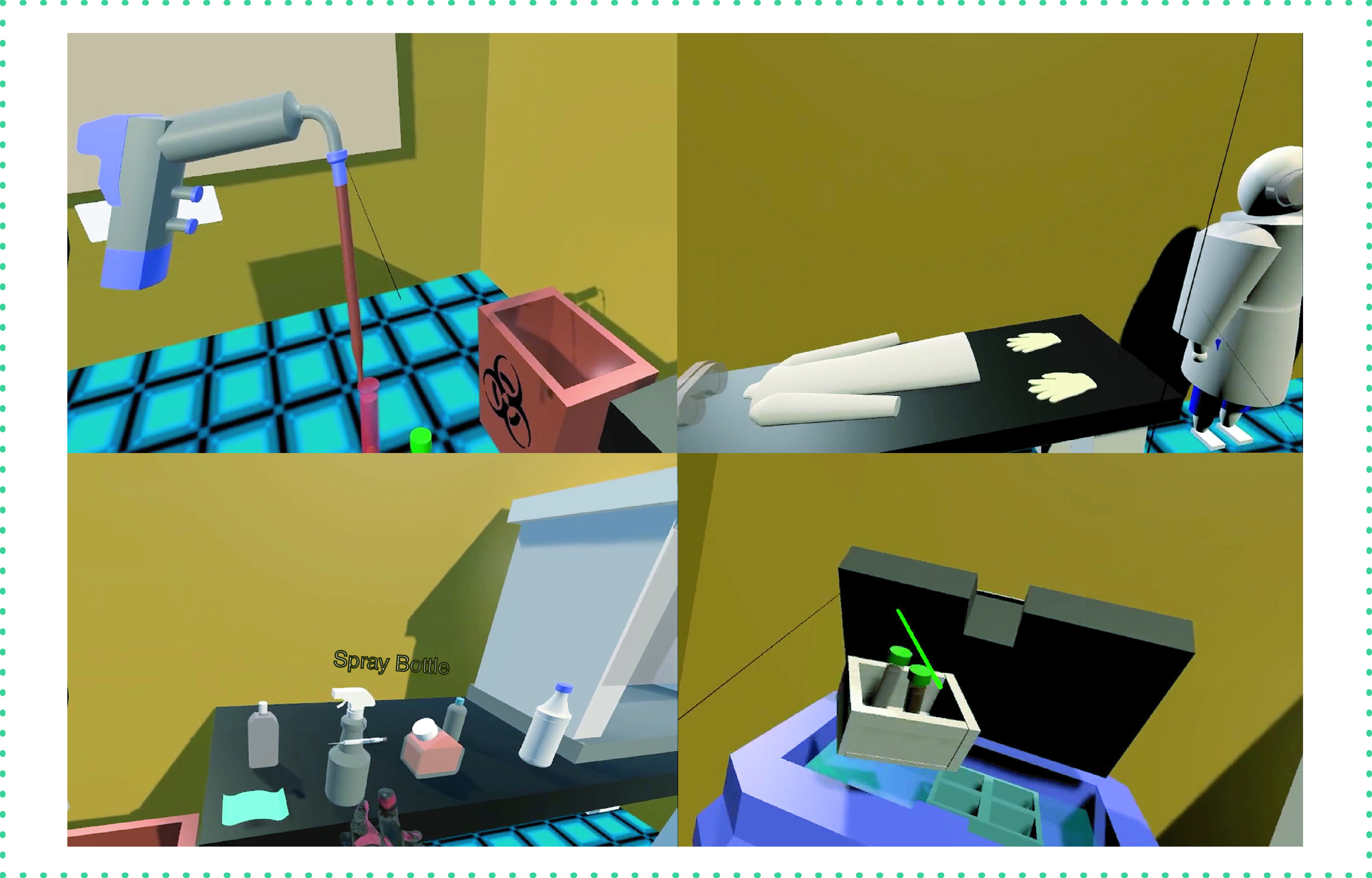
Cell culture virtual reality app. Virtual reality app for training students in cell culture methods, including sterile techniques, use of personal protective equipment and culturing. The app simulates a cell culture laboratory with tasks requiring students to practice sterile techniques, use of personal protective equipment, use of laboratory equipment and cell culturing. Much of the laboratory equipment is interactable (e.g., pipettes can take and expel liquids, cabinets can be opened and closed, containers can be opened, etc.). Interactive quizzes are used to test students' knowledge and allow them to proceed through the simulation.

Barriers to providing hands-on training on aseptic cell culture techniques include a lack of sufficient workspaces to train large numbers of students, the time-intensive nature of the training and the laborious process of identifying and containing cell culture contamination. The app mimics a modern cell culture laboratory for students to learn the techniques in a safe environment. Once familiar with the procedures, students can transition to wet lab training (Figure 5). Rooms in the app introduce students to personal protective equipment, aseptic techniques and cell culture methods. These rooms allow the students to interact with different components commonly found in a cell culture laboratory, including the biological safety cabinet, cell culture incubator, centrifuge, light microscope and liquid nitrogen tank for cryostorage. A whiteboard containing instructions is in all rooms and is used to quiz the students on important concepts. Essential procedures taught include trypsinization, passaging, pelleting, cell counting and thawing frozen cells from the liquid nitrogen tank. This was the first app tested for cloud deployment using Amazon Web Service (AWS) and Amazon AppStream.

#### Nanoparticle & liposome VR

**Figure 6.**
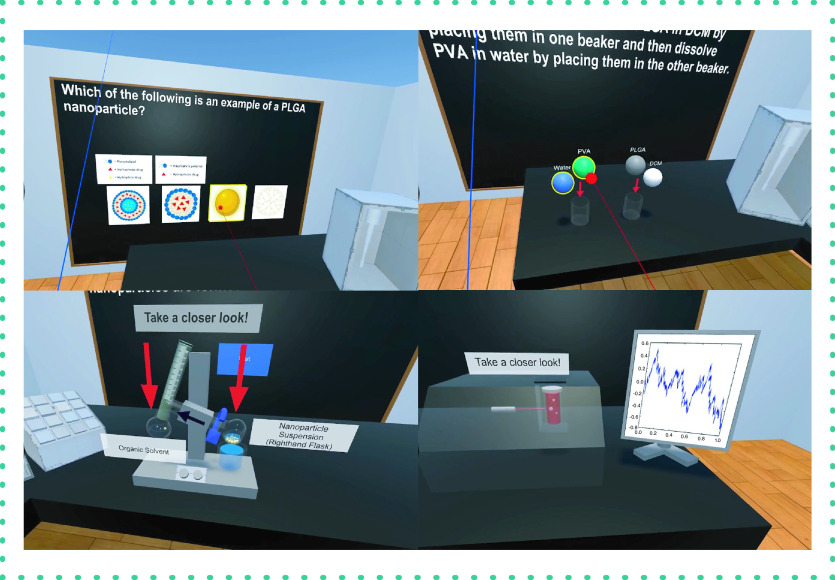
Nanoparticle virtual reality app. Virtual reality app for teaching concepts in nanoparticle synthesis. The app simulates an experimental laboratory using virtual equipment used to produce nanoparticles. Interactive quizzes are used to test students' knowledge and allow them to proceed through the simulation. Steps include understanding the chemical nature of nanoparticles, synthesis of nanoparticles using standard laboratory equipment and evaluation of the resulting nanoparticles.

The second EVEREST project led by Jie Shen focused on a pair of related apps covering the creation of nanoparticles and liposomes (Figure 6). Nanoparticles are used in many modern drug delivery methods due to their ability to target specific tissues and control therapy release. The nanoparticle VR apps recreate visually accurate laboratories with equipment such as the rotary evaporator, sonicator and zetasizer required for the synthesis and characterization of nanoparticles and liposomes. Students are quizzed on various aspects of nanoparticle chemistry and synthesis.

### Year 3 apps

Current projects are based on previous ILT projects with the goal of either updating them to a VR format or upgrading them from the proof-of-concept phase to more sophisticated versions that can better be incorporated into the COP curriculum. One such example is the Diuretics VR app, which is based on a prior collaboration between ILT and Roberta King (COP) to visualize ion transport in the kidney nephron and the molecular mechanism of action of commonly used diuretic drugs. Users are placed in a virtual nephron inside three specific cell types in which ion transporters are located. Using guided tutorials, users interact with the ion transporter proteins by feeding them ions (Na^+^, K^+^ and Cl^-^) to see the effects of ion transport in nephrons.

### Technical challenges

Developing software in academic settings is difficult because the institutional knowledge of professional software development companies often does not exist. Part of the process of developing these apps includes building institutional knowledge within ILT and MIC to successively improve the apps. One issue encountered early in the process involved rendering and coloring protein structures. Initially, one goal was to conserve computational resources by first coloring molecules in Chimera, then saving them as 3D objects before importing them into Unity. This process stripped away the coloring of the molecules due to a convention in molecular visualization that utilizes vertex coloring, which is ignored by most Unity shaders. Some molecules were then colored internally or UnityMol was used. Another development was the use of custom-designed 3D objects. In the initial year 1 apps, publicly available 3D models for in-app objects were mostly used, but this was not always ideal, as models for scientific instruments are often inaccurate or “cartoony.” Thus, specific building models (e.g., water baths, pipettes, distillation columns, etc.), allow more scientifically accurate content. This allowed a better simulation of a “real” scientific laboratory so that students could easily associate equipment in virtual laboratories with real equipment in physical laboratories. AR presented challenges in terms of computational load on mobile devices and screen visualization when used outdoors, which required a redesign of the interface and background processes. A more complete list of technical challenges and solutions is shown in Supplementary Table 1.

### Assessment of applications

While formal educational assessments of the apps have not been conducted, informal pre- and post-use surveys of the MTX VR and nanoparticle VR apps to determine their utility in STEM education were conducted. The MTX VR app was assessed by a large group (n = 117) of COP fifth-year pharmacy students in the oncology class (BPS 521) on their opinions of the app's effectiveness before and after experiencing VR simulation. For the presurveys (n = 115), students agreed (52%) that the VR simulation might benefit learning the MTX mechanisms. The number increased dramatically (96%) in the postsurvey (n = 117), and students felt that they had confidence in their understanding of MTX's anticancer molecular mode of action. For nanoparticles VR, 14 students (only two of whom had prior experience with VR) were surveyed before and after the use of the app. On a scale of 1 (strongly disagree) to 5 (strongly agree), students were asked prior to the use of the app if they understood the process of creating and characterizing nanoparticles (2.86) and if they felt confident explaining how nanoparticles are formed and characterized to a peer (2.56). These averages increased to 4.64 and 4.36, respectively, following the use of the VR app. Students were also asked post-use if they felt that the VR app could enable students to practice hands-on laboratory skills (4.71) if the 3D gaming app could enable students to practice hands-on laboratory skills (4.57) and if virtual tools in teaching could help student learning (4.64). The overall sentiment of the students regarding the app was very positive.

## Conclusion

The collaboration between COP, ILT and RI-INBRE has resulted in numerous VR/AR apps that are being used in the URI COP curriculum. The use of VR/AR and 3D gaming apps allow for a broader development of tools for teaching pharmacy concepts and greater institutional knowledge among ILT students, allowing for the creation of more sophisticated apps. Utilizing the ILT program allows for the development of apps covering a broad scope of topics beyond the pharmaceutical sciences. We hope to develop apps outside the scope of pharmaceutical sciences and build partnerships with other Rhode Island universities in the RI-INBRE network. Continued assessment of these apps and their users will allow us to refine our production pipelines further to ensure that students are receiving the maximum benefit from using these apps.

These apps are also vital for outreach to the Rhode Island community, such as visiting high school students interested in STEM topics, recruitment of prospective students for the College of Pharmacy, the RI National Guard STEM Open House 2022 (with the URI College of Engineering) and the Global Science & Envirotech summer outreach program for RI middle and high school students. We will build on our institutional knowledge of MR apps to create more innovative and sophisticated apps for STEM research and outreach. For ILT, student assistants are challenged to learn new skills that will aid them in their future job prospects. For RI-INBRE, the development of these apps is an essential part of the MIC 3D visualization component and has provided RI-INBRE researchers with additional mechanisms for pursuing their research. Future goals are described next.

### Virtual room capability

We are exploring ways to make the apps accessible to students in either VR or 3D gaming mode through AWS AppStream. Ideally, we would like to replicate the virtual room capability of apps such as Nanome to allow for remote collaborative use of the apps, significantly improving accessibility and convenience of deploying apps in a classroom setting.

### Expansion beyond pharmacy

Most of our apps are focused on pharmaceutical sciences. We want to expand the scope of apps to other colleges at URI, such as the College of Environmental and Life Sciences, the College of Engineering and the Graduate School of Oceanography, and to the primarily undergraduate institutions (PUIs) of the RI-INBRE network. We are discussing partnering PUI faculty with URI faculty to create shared VR/AR/3D gaming resources for use in curriculum at multiple institutions.

### Mixed reality

While most of our apps have focused on VR, an MR strategy that takes full advantage of all available technologies would better position us to meet the demands of our user base. This strategy will require the acquisition of additional technologies such as Microsoft Hololens or zSpace hardware, as well as emerging technologies such as wearable AR/MR devices.

## Future perspectives

The popularity of MR has waxed and waned, but the current implementation of these technologies appears to have more staying power due to cheap mobile and wearable devices, greater computing power to render more realistic environments and user-friendly development platforms. These technologies have democratized MR app development. While the promise of wearable MR devices and concepts such as the “metaverse” have not always lived up to their promise, we expect cheap and comfortable wearable MR devices will eventually become the norm. Advances in cloud computing allow much of the computational load of an app to be offloaded to the cloud environment, allowing for the creation of more powerful and sophisticated apps. Generative artificial intelligence (AI) will also likely become an important component of the MR app development process. Already, text-based generative AI systems such as ChatGPT are used to generate code and blocks of text, and visualization-generative AI systems such as Midjourney could be used to quickly generate virtual content. These technologies are expected to further democratize the process of app development, allowing users to quickly create apps even if they lack a formal programming background.

**Executive summary**

- This work provides an introduction to the use of virtual and augmented reality (VR/AR) applications in the biomedical sciences and the rationale for the development of VR/AR apps at the University of Rhode Island College of Pharmacy.

**Materials & methods**

- Methods and tools used for the development of VR/AR apps including the hardware used and the software development environment are described.

**Results**

- Discussion of specific VR/AR apps developed at the University of Rhode Island and the iterative approach to improvement of the development process are also discussed. The developed apps include:
  - Aspirin VR.
  - Methotrexate VR.
  - Natural products VR.
  - Medicinal Garden AR.
  - Cell culture VR.
  - Nanoparticles VR.
  - Nephron VR.

**Technical challenges**

- Technical challenges encountered in the development of the apps included creating a functional and comfortable user interface, displaying complex scientific topics such as biochemical pathways in VR, and maximizing accessibility of the apps, particularly for users unable to use stand-alone VR equipment.

**Assessment of applications**

- Assessment of the utility of the apps by student testers for improving science, technology, engineering and math education is elucidated.

**Conclusion**

- Future development projects and tasks such as the inclusion of virtual rooms and improved accessibility are proposed.

**Future perspectives**

- The current and future utility of using VR/AR in science, technology, engineering and math education including the role of cloud computing and generative artificial intelligence are discussed.
