# Supplementary material for: Developing virtual and augmented reality applications for science, technology, engineering and math education
Source: Biotechniques. 2023 Jun 9;75(1):11–20. doi: 10.2144/btn-2023-0029 (PMC10505987; doi:10.2144/btn-2023-0029)
Supplement: Supplementary file 1 [file btn-75-11-s1.docx]

**Supplementary Table 1. Technical challenges in development of academic VR/AR apps.**

| Application | Challenge | Solution |
| --- | --- | --- |
| Aspirin | Rendering of biochemical pathways in VR in a traditional left-to-right fashion was sub-optimal. | We rotated the pathway into the third dimension, aligned and resized the molecular structures, and then selectively turned layers on and off to create a molecular slide show. |
| Aspirin | In early versions of the app, the user interface suffered from disproportioned objects compared to the player, resulting in disorientation. | With experience, we became more adept at properly sizing objects in Unity, reducing this effect. We also began the practice of adding textures to plain walls/floors to create perspective and eliminate a “sinking effect” when in VR. |
| Aspirin | To save computational power, we imported and colored protein structures in Chimera and saved them as 3D objects, but the coloring was lost when importing into Unity. | 3D visualization of molecules typically saves the coloring information as vertex colors, which are usually ignored by Unity shaders. Coloring can be manually applied in Unity or handled by software such as UnityMol. |
| Methotrexate | The Aspirin app used a great deal of text and was too wordy. | We switched to using text-to-voice functionality to reduce the amount of reading required. Aside from a few minor mispronunciations of scientific terms, this worked very well. |
| Methotrexate | We needed a way to quickly convey information to the user in app and to assess their progress. | We developed in-app whiteboards to instruct users and to quiz them on concepts. |
| Methotrexate and later apps | Large “museum” spaces such as in the Aspirin app were not always necessary and could cause significant use of computational resources. | We switched to small, focused rooms linked by teleportation (i.e. complete quiz and move to next room). This saved computational resources and was less overwhelming to users. |
| Cell Culture/Nanoparticle | Demonstrating VR apps using the headsets was only feasible for small groups of students. | To improve accessibility and throughput for larger groups of students, we developed 3D gaming versions of the apps that did not require VR headsets. We also made the apps available through AWS AppStream. |
| Cell Culture/Nanoparticle | Freely available 3D models were not sufficient to accurately portray a modern biochemical laboratory. | We began constructing our own 3D objects, including interactive appliances (refrigerators, water baths, etc.) and lab equipment (distillation column, microscopes, etc.) |
| Cell Culture | Keeping track of the progress of tasks in each room presented a challenge in being both complex and still trying to be readable | A generalized task manager was created to abstract the text storage of each individual task. Once a task manager completes all its tasks it then starts the quizzing system which runs through a multiple-choice quiz then allows for the user to move on to the next room. |
| Cell Culture | During early testing a common issue that players would run into is that often in VR items would get knocked over and would often fall to the ground, so this made it difficult for the object to be picked up and often required the user to restart. | This was resolved by introducing a system where any object that could be picked up and moved around would store the position and rotation it started in then if at any point the object detects that it is touching the ground it will reset itself back to the original position and rotation. |
| Cell Culture | For a large amount of time if a held object would hit another object it would start to rotate uncontrollably, and the user would be unable to use the object properly. | For this there were two main fixes that were used. The first fix was to allow the user to press space at any time while holding an object to reset its rotation. The second fix was to prevent the object's rigid body from updating any physics while being held by freezing its rotation. |
| Nephron | Design wise the previous project (Cell Culture App) was built continuously where objects were added as they were needed would often lead to a large amount of restructuring and time spent ensuring that the new objects would function properly while also building a section of the application. | The Nephron App design took on an object focused approach where development initially focused on creating and building objects that would be used in the application and preparing and testing them ahead of time to streamline the process of building rooms and creating the experience. |
| Nephron | The Ion objects were scattered on the floor making it difficult for users to properly interact with and pass them through the proper transport proteins, | The ability for the player to point at an ion and click on it to have it slowly move towards the player was added to make it easier for the player to grab without bending down. Transport proteins were changed so that if any ions were placed into the protein, the rest of the ions in that group automatically moved into and through the protein. This simplified the process of picking up and placing ions while still conveying the number of ions that would pass through the transport protein |
| Nephron | During testing players found it difficult to find the proper protein to place the ion objects into and struggled to find the proper place within the protein to place them. | For each task in a room the proper protein changed color to "highlight" it and make it easier to spot. The pipe that acted as the detection for ions was also highlighted to make it easier to spot and place ions into. Small rug objects would also appear in front of the players to help show them where they should stand to properly place the ions into the protein. |
| Garden AR | We originally intended to use QR codes to identify the individual plants, but this introduced computational challenges and was not aesthetically pleasing. | We created laser-etched wooden signs with high contrast etching (better for outdoor use) which are scanned by the app. This works as well as QR codes and is more aesthetically pleasing in the garden. |
| Garden AR | The original version of the app was difficult to use outside (particularly on sunny days) and required users to stand in uncomfortable positions to view the molecules in AR. | We redesigned the user interface based on user feedback to make it more readable outdoors and less wordy. To better allow users to view the molecules, we uncoupled the molecule from the location of the scanned plant sign (i.e. users no longer have to stand over the sign to see the molecule). |
